# Shared IVIVR for Five Commercial Enabling Formulations Using the BiPHa+ Biphasic Dissolution Assay

**DOI:** 10.3390/pharmaceutics13020285

**Published:** 2021-02-22

**Authors:** Alexander Denninger, Ulrich Westedt, Karl G. Wagner

**Affiliations:** 1 Department of Pharmaceutical Technology, University of Bonn, Gerhard-Domagk-Straße 3, 53121 Bonn, Germany; alexander.denninger@uni-bonn.de; 2 AbbVie Deutschland GmbH & Co. KG, Knollstrasse, D-67061 Ludwigshafen, Germany; ulrich.westedt@abbvie.com

**Keywords:** biphasic dissolution, poorly soluble drugs, enabling formulation, IVIVR, biopharmaceutics, drug product development, formulations screening

## Abstract

The present study intended to confirm the in vivo relevance of the BiPHa+ biphasic dissolution assay using a single set of assay parameters. Herein, we evaluated five commercial drug products formulated by various enabling formulation principles under fasted conditions using the BiPHa+ assay. The in vitro partitioning profiles in the organic phase were compared with human pharmacokinetic data obtained from literature. In the first part, a meaningful in vitro dose of the formulations was assessed by determining the maximum drug concentration in the artificial absorption sink during dissolution (organic 1-decanol layer, *C_dec,max_*). Then, the maximum concentration of the partitioned drug in the organic layer was correlated with the in vivo fraction absorbed, which was derived from published human pharmacokinetic data. Fraction absorbed represents the percentage, which is absorbed from the intestine without considering first pass. It was found that the maximum drug concentration in the organic phase obtained from an in vitro dose of ten milligrams, which is equivalent to 15–25 µmol of the respective drug, led to the highest congruency with the fraction absorbed in vivo. In the second part, the in vivo relevance of the BiPHa+ dissolution data was verified by establishing a shared in vitro/in vivo relationship including all formulations. Based on the in vitro kinetics of the BiPHa+ experiments human in vivo plasma profiles were predicted using convolutional modelling approach. Subsequently, the calculated pharmacokinetic profiles were compared with in vivo performance of the studied drug products to assess the predictive power of the BiPHa+ assay. The BiPHa+ assay demonstrated biorelevance for the investigated in vitro partitioning profiles using a single set of assay parameters, which was verified based on human pharmacokinetic data of the five drug products.

## 1. Introduction

Ideally, dissolution methods used in early stages of formulation development should be appropriately predictive of the in vivo performance of the formulation. For this purpose, the in vivo relevance can be confirmed by the development of different stages of in vivo/in vitro relationships (IVIVR) [1,2]. Based on the approved regulatory guidelines, sink conditions are obligatory to characterize the drug release from the formulation. For a BCS I compound it is assumed that the drug release from the dosage form represents the rate-limiting step, whereas dissolution is the rate-limiting step for BCS II/IV compounds. Because of the limited solubility of BCS class II and IV drugs in aqueous media, the discrimination regarding the in vivo performance is sometimes not sufficient by applying compendial methods. The generation of sink conditions for these challenging drugs is often achieved through the addition of surfactants or co-solvents, or by using large amounts of solvent [3]. However, these modifications have not led to a ground-breaking success in terms of establishing a suitable dissolution assay for poorly water-soluble drug compounds [3].

Therefore, efforts have been made to overcome such limitations through the development of biorelevant dissolution methods, such as biphasic dissolution systems [3,4]. In this technique, the drug product is initially placed in the aqueous dissolution layer under non-sink conditions. Then, an absorption-sink is generated by layering an organic solvent on the aqueous medium, which is assumed to be more discriminatory or predictive to in vivo [5]. To date, several studies have demonstrated in vivo relevance of the biphasic dissolution assay for various drugs by establishing level A IVIVRs [4,6,7,8,9]. However, the correlations have not been evaluated in a consistent way, as either the drug concentration time profile in the organic layer alone, or in combination with the concentration time profile in the aqueous layer were used to establish the IVIVR. Additionally, the test parameters were mostly adapted to optimally fit formulations of a distinct drug, rather than proposing general assay parameters of formulations of various drugs.

Hence, the aim of the present study was to assess and verify the relevance of the biphasic partitioning profiles of different commercial drug products for their passive gastrointestinal absorption behavior in vivo using a single set of assay parameters. The selection of drug products comprised microcrystal, nanocrystal, and amorphous solid dispersion (ASD) formulations. Fasted state conditions were assumed as worst case, because of the higher intestinal pH value compared to fed state conditions, which lead to a decreasing solubility for weakly basic compounds, and lower surfactant concentration in the intestine, which often leads to an essentially lower solubility capacity compared to fed state media [10].

Firstly, the effect of the formulation sample quantity (in vitro dose) was investigated with respect to the maximum drug concentration reached in the organic layer (*C_dec,max_*) at the end of the dissolution experiment, to find the most bioequivalent settings. For this, the relative *C_dec,max_* of the partitioned drug was compared to the fraction absorbed in vivo, which was derived from published human pharmacokinetic data, to find a bioequivalent in vitro dose. The fraction absorbed represents the percentage, which is absorbed from the intestine without considering the first pass [11].

Secondly, the human pharmacokinetic data were analyzed using the residual method to obtain the kinetic parameters for drug absorption, distribution, and elimination [12].

Finally, the biorelevance of our biphasic dissolution assay BiPHa+ [13] was compared with the in vivo data by taking the following steps: An in vitro/in vivo relationship (IVIVR) for each of the five model formulations was established and in vitro partitioning profiles were directly compared to in vivo absorption profiles. Subsequently, obtained in vitro partitioning data were convoluted into predicted in vivo plasma concentration time profiles according to the compartment model. The rate constants of one or two compartment models [12] were calculated based on in vivo literature data. Subsequently, the predicted in vivo plasma concentration time profiles were compared to the observed plasma concentration time profiles.

## 2. Materials and Methods

### 2.1. Materials

The selected commercial drug products were purchased from a public pharmacy (Table 1). Hard gelatin capsules size 4 (Capsula GmbH, Ratingen, Germany) were used for those formulations, which represented pellets or powders. Tablet formulations were dose proportionally cut with a knife to create the respective in vitro dose (Table 1). The buffer concentrate contained tri-potassium phosphate (Alfa Aesar, Kandel, Germany), tri-potassium citrate (Carl Roth, Karlsruhe, Germany), and sodium hydroxide (VWR Chemicals, Darmstadt, Germany). Sodium-taurocholate, lecithin and 1-decanol were purchased from Alfa Aesar (Kandel, Germany). The high performance liquid chromatography HPLC chemicals consisted of methanol (VWR Chemicals, Darmstadt, Germany), and demineralized water (Merck Milli-Q, Darmstadt, Germany). 0.1 N hydrochloric acid was purchased from VWR Chemicals (Darmstadt, Germany). The following drug substances were used: aprepitant (TCI Deutschland, Eschborn, Germany), celecoxib (Acros Organics, Thermo Fisher Scientific, Geel, Belgium), itraconazole (Acros Organics, Thermo Fisher Scientific, Geel, Belgium), nimodipine (Alphar aesar, Landau, Germany) and ritonavir (AbbVie Deutschland GmbH & Co. KG, Ludwigshafen, Germany).

### 2.2. Physiochemical Characterisation

pKa values were determined through UV-metric titration using a Sirius-T3 apparatus (Pion Inc (UK) Ltd., Forest Row, UK). Pure drug substance of aprepitant, celecoxib, itraconazole, nimodipine, and ritonavir were titrated from pH 1 to 11. The pKa values were determined at the maximum change of UV-Vis spectrum [14].

A HPLC method with a water-methanol gradient was used for the LogP measurements on a HPLC-DAD system (Waters, Eschborn, Germany) using a Lichrospher RP-18 5 µm 10 × 4 mm column [15].

Shaking flask method was applied for the determination of the thermodynamic solubility of pure drug substances in 0.1 N HCl, 6.8 buffer, FaSSIF-V2 and in 1-decanol for 72 h at 37 °C. All samples were centrifuged for 20 min at 4500 rpm (r = 147 mm, relative centrifugal force = 3328). The supernatant was analyzed by the same HPLC method as used for LogP [15] to determine the concentration in FaSSIF-V2. The samples obtained in 0.1 N HCl, phosphate buffer pH 6.8 and in 1-decanol were directly quantified by an Agilent 8453 UV-Vis spectrometer (Agilent, Waldbronn, Germany).

### 2.3. Biphasic Dissolution Assay

**Figure 1:**
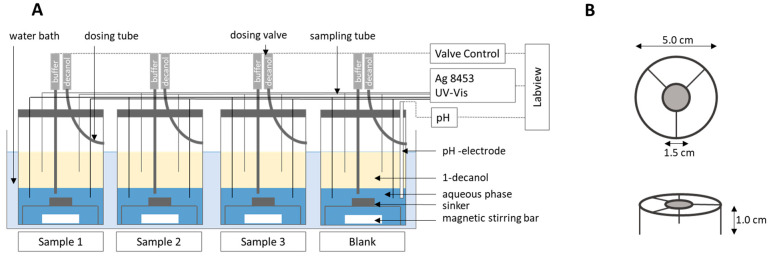
(**A**) Biphasic dissolution assay setup, (**B**) sinker dimension. Adapted from [13], Pharmaceutics, 2020.

All formulations (Table 1) were investigated by the fully automated BiPHa+ apparatus in cylindrical vessels with a diameter of 5 cm (Figure 1). The experiments in triplicate (n = 3) were carried out in parallel. Homogenous mixing was achieved by using triangular magnetic stirrers. A detailed description of the method development is provided in our previous work [13]. All formulations were placed in a sinker above the stirrer. Tablet formulations were adjusted to the desired drug dose strength with a knife. Pellets and powders were filled in hard gelatin capsules and placed into the sinker (mesh size 1 mm) [13].

The experimental sequence was designed to mimic the gastrointestinal passage in a fasted state. pH profile, bile-surfactant concentration and transition time were adapted to in vivo fasted state conditions forming the Bi-FaSSiF-V2 buffer [13,16]. The formulations were placed into the dissolution vessel with 50 mL of a 1-decanol pre-saturated 0.1 N hydrochloric acid solution for 30 min. Then, the pH was adjusted to intestinal pH of 5.5 by adding a buffer concentrate together with an aqueous Bi-FaSSIF-V2 surfactant concentrate [13,17]. By this, the aqueous mixture exhibited comparable properties in terms of osmolarity, buffer-capacity and bile salt surfactant concentration to in vivo fasted conditions [14,18,19,20]. Dissolution in the aqueous phase follows a physiological pH trajectory. Latest at the pH shift to pH 5.5, non-sink conditions were generated; i.e., particulate matter dependent on its solid state will affect re-dissolution and distribution into the organic phase. This is of vital importance, since biorelevant conditions for poorly soluble active ingredients are intended to be simulated with the applied assay. Subsequently, the aqueous layer was covered with 50 mL of water-saturated 1-decanol as an absorption sink compartment. The respective 1-decanol volume was selected to create a more than 5-fold sink condition for all drugs. For that reason, the 1-decanol volume was increased to 100 mL for itraconazole. The 1-decanol absorption layer is intended to simulate the potential absorption of a drug substance during the intestinal transit. Koziolek et al. reported the small intestine transition time under fasted conditions as a period of 240 min [16]. After 90 min, the pH was stepwise adjusted to 6.8 and was kept on the same level until the end of the experiment. The mean gastric and small intestine transition time under fasted conditions, which is similar to our experimental duration, was 270 min [16].

The concentration profiles were measured online by using an Agilent 8454 UV-Vis spectrometer (Waldbronn, Germany) equipped with 0.1 cm flow through cuvettes. Aqueous and organic phase were continuously circulated through 0.1 cm flow cuvettes and back into the vessel. For reduction of precipitated material in the flow through cuvettes of the aqueous phase, a 1 µm full-flow filter was employed. All experiments were carried out parallelly in triplicate. The measured spectra were processed by an exponential correction or by calculating the derivative to exclude scattering or overlapping of spectra (Table 3). The online measurement, quantification and correction of spectra were conducted by using a programmed LabView^®^ software for the BiPHa+ dissolution test [13].

### 2.4. Dose Assessment

The aim of the present study was to find model settings, which are generally applicable for formulation approach assessment even if the potential clinical dose is not known. Therefore, the effect of the formulation quantity (in vitro dose) was investigated regarding the obtained partitioning profiles in the organic layer. Three formulation quantities were selected referring to an in vitro dose of 5 mg, 10 mg, and a BCS class dependent quantity (Table 1). To calculate the BCS dependent quantity, the highest dose on the market was divided by 250 mL and multiplied with 50 mL [13,21].

As some experiments resulted in small partitioned drug quantities in the organic phase for some formulations (Section 3.2), we empirically selected next to the BCS dependent quantity two in vitro doses, namely 5 mg and 10 mg. We assumed a higher relative partitioned drug quantity (relative *C_dec,max_*) in the organic absorption sink layer by decreasing the in vitro dose. The rational for the in vitro dose selected is based on the following considerations: The organic phase (absorption sink) pulls dissolved drug molecules out of the aqueous phase. Since only the dissolved drug substance is able to partition into the organic phase, a minimum aqueous solubility of undissolved drug particles, dispersed in the aqueous phase (crystals, precipitated particles (amorphous or crystalline), LLPS, colloids), is essential. The undissolved drug particles form the reservoir for the molecularly dissolved drug substance to be distributed into the organic phase. The extent of the reservoir is linked to the in vitro dose used in the biphasic assay. Moreover, Locher et al. 2016 proved a dissolved drug concentration dependent mass transfer [22]. Additionally, mass transfer is a function of interfacial area (aqueous/organic), hydrodynamics and the extent of sink in the organic phase [23]. Now, it is important to balance drug reservoir (in vitro dose) and mass transfer. It was hence strongly anticipated that an optimal in vitro dose would result in distribution kinetics optimally matching in vivo absorption kinetics and consequently assessed experimentally.

The in vivo fractions absorbed for each formulation were obtained from literature and compared to the maximum concentration at the end of the experiment (relative *C_dec,max_*) from the 5 mg, 10 mg, and the BCS-class dependent quantity (Table 1). Fraction absorbed (f_a_) is the absorbed quantity without considering metabolic pathways. Bioavailability (F) considers metabolism during absorption. A high degree of biorelevance is assumed, when relative *C_dec,max_* values (percentage drug in the organic layer in %) are comparable with the in vivo fractions absorbed [4,8].

### 2.5. Pharmacokinetics and Compartment Analysis of In Vivo Data

**Figure 2:**
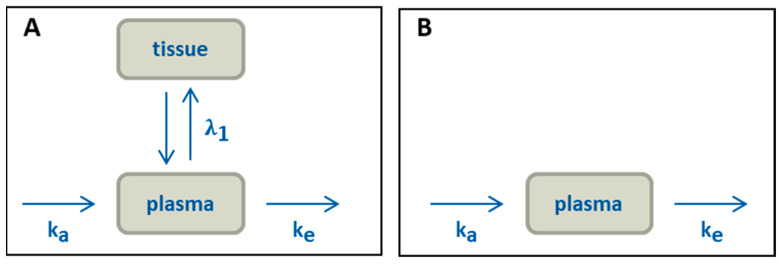
Pharmacokinetic models: (**A**) two-compartment model, (**B**) simplified model for aprepitant.

All pharmacokinetic profiles were evaluated using an oral two-compartmental model (Figure 2A). Given that the LogP values of the evaluated drugs were higher than 3 (Table 3), a two-compartment model was applied to accurately describe the in vivo behaviour, which includes a distribution step of the drug in a peripheral compartment. [24,25].

**Figure 3:**
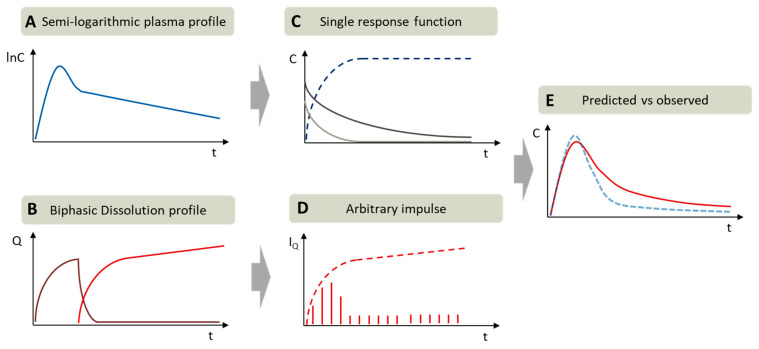
Exemplary procedure of pharmacokinetic parametrization by residual method (curve stripping) and in vivo prediction assessment: (**A**) Semi-logarithmic plasma concentration time profile; (**B**) biphasic dissolution profile: aqueous phase (purple) and organic phase (red); (**C**) first order absorption (dark blue) and the determined single impulse functions: distribution (light grey), elimination (dark grey) received by residual method; (**D**) Arbitrary impulses (*I_Q_*) calculated by in vitro partitioning rate on each time point (red stripes), (**E**) comparison of predicted plasma profile (red) vs. observed plasma profile (blue).

The sub-processes of the two-compartmental model, absorption ($\;C\;{\left(t\right)}_{absorption}$), distribution ($A\;\cdot C\;{\left(t\right)}_{distribution}$), and elimination ($B\cdot C\;{\left(t\right)}_{elimination}$), were assumed to show first order kinetics (Equation 1). The kinetic rate constants of all drugs were obtained by the residual method [24,26]. For this, the observed plasma profile data were linearized by calculating the logarithm of plasma concentration over time (Figure 3A). At first, the first order elimination functions ($B\cdot C\;{\left(t\right)}_{elimination}$) were calculated and subtracted from the observed plasma data. Then, the obtained data points were used to calculate the first order distribution function ($A\;\cdot C\;{\left(t\right)}_{distribution}$). Factor *A* and *B* were the intercept of the determined elimination and distribution functions and could be interpreted as scaling factor towards the absorption function ($\;C\;{\left(t\right)}_{absorption}$). For all calculations, distribution was assumed to be slower than absorption. Therefore, absorption was calculated by subtracting distribution and elimination function from the plasma data. The absorption rate constants of $C\;{\left(t\right)}_{absorption}$ were estimated from the logarithm of the residual concentration values.

In addition, the fraction absorbed (f_a_) was needed to assess the biorelevance of the partitioning profiles (Section 2.4). A high degree of biorelevance was assumed if *C_dec,max_* values of the organic phase and fraction absorbed values were equivalent [4,8].

All calculated absorption profiles were assumed to show first order kinetic without knowing the real in vivo kinetic [24,26]. However, this calculation method is useful to get an idea about the amount and kinetic of the absorption process in vivo.

(1) $$C\left(t\right)=\;C\;{\left(t\right)}_{absorption}-A\;\cdot C\;{\left(t\right)}_{distribution}-B\cdot C\;{\left(t\right)}_{elimination}$$

Aprepitant (Emend^®^, Nanocrystal)

The oral pharmacokinetics of the market formulation was assessed in two clinical studies by Majumdar et al. From the first study the absolute bioavailability of 59% has been reported based on the data obtained from 20 volunteers under fasted conditions [27]. The second study provides the pharmacokinetics based on the data obtained from 21 volunteers, assuming a one-compartment model [28]. Analysis of the study was performed by the residual method analysis of the fasted state plasma profile (Figure 2B), because of the inhomogeneous elimination period (10 h – 40 h) of aprepitant. The inhomogeneous elimination is apparent by the slower elimination rate between 10 and 20 hours compared to the later increased elimination rate (Figure 8A).

Celecoxib (Celebrex^®^, Microcrystal)

The plasma profiles reported by Paulson et al. [29] were utilized for the pharmacokinetic evaluations of the two-compartment model in the present work (Figure 2A). It should be noted that the absolute bioavailability of celecoxib in human has not been determined, since no intravenous formulation of the drug has been developed and tested so far [30]. In the study by Paulson et al., the absolute bioavailability of micronized celecoxib of approximately 40% was obtained from a study in poor metabolizing beagle dogs under fasting conditions [29]. In this case, minimal first pass effects were expected, and the fraction absorbed was assumed to be the maximum amount of passive absorbable drug, which was correlated to the results of the biphasic dissolution model.

Itraconazole (Sempera^®^, ASD)

Brone et al. investigated the plasma profile of 100 mg itraconazole in healthy male volunteers [31]. The absolute bioavailability of the oral solution was examined at fasted state [32]. The relative bioavailability for capsule and solution were compared to calculate the oral absolute bioavailability of the capsule formulation [33]. The absolute bioavailability was reported to be 16%. A two-compartmental model was assumed to evaluate pharmacokinetics (Figure 2A).

Nimodipine (Nimotop^®^, ASD)

The pharmacokinetic data were taken from Blardi et al. [34] Nimodipine undergoes a strong first-pass effect and hepatic metabolism of about 85–95% resulting in a low bioavailability of 3.5% when taken under fasted condition [34]. Without first-pass, the real fraction absorbed could be around 35% based on a mean metabolizing value of 90% [35]. Because of the very fast metabolism (much faster than absorption), the metabolic rate can only be calculated imprecisely with the residual method and is not relevant for the kinetic of the absorption [24]. Consequently, pharmacokinetic was evaluated based on a two-compartmental model (Figure 2A).

Ritonavir (Norvir^®^, ASD)

Klein et al. investigated the pharmacokinetic of 100 mg Norvir^®^ in 27 healthy human subjects [36]. Xu et al. established a level A IVIVR with different ritonavir drug products including a fraction absorbed of 60–80% [8]. They suggested a direct correlation between the maximum fraction absorbed and the maximum partitioned drug substance, in this case approximately 75% [8], which was used in the present work. A two-compartment model was applied to calculate the rate constants (Figure 2A).

### 2.6. Verification of In Vivo Relevance and Predictive Power

The biphasic partitioning profiles of the five model drugs formulations were assessed with respect to their in vivo relevance. A level A IVIVR was calculated, partitioning and absorption profiles were directly compared. Prediction errors were calculated comparing the predicted and observed plasma concentration time profiles. As many mathematical methods were performed, we illustrated the methods applied for in vitro and in vivo data treatment in Table 2.

As described in Section 2.5, two-compartment models were applied to gain the in vivo fraction absorbed versus time profiles (Figure 3). The rate constants of absorption, distribution and elimination were calculated by the residual method [24,26] using data obtained from the literature (Figure 3A,C). The first order absorption model was selected, because the deconvolution approach leads sometimes to a limited number of data points (drug concentration time values) in the in vivo absorption period which can be respectively correlated to in vitro data [24,26].

In a first step, the calculated in vivo absorption profiles and the in vitro partitioning profiles (drug concentration over time in the organic layer) were used to calculate a level A IVIVR [37,38] as follows:

(1) A Levy-plot (normalization of time scale) was established for all five formulations to estimate a general time-scaling factor between in vitro and in vivo data [39].
(2) The level A IVIVR was established by applying the Levy-plot (Section 3.5.1).

In a second step, the drug concentration time profiles of the organic layer were compared to those obtained by absorption in vivo in the same figure (Section 3.5.1).

For an assessment of the BiPHa+ data compared to the in vivo pharmacokinetic, retro-IVIVRs (single stage [1]) were calculated based on a convolutional approach, where *C_δ_*(*t*) is the impulse response and *I_Q_*(*t*) are the arbitrary impulses which can be interpreted as the amount of released/absorbed drug [40]. The arbitrary impulses are the first derivative of the dissolution profile, calculated by change of drug concentration in the organic phase at each measured time point. The predicted plasma concentration time profiles are the sum of all unit impulse responses (*C*(*t*) vs. *t*, Equation (2)). Each impulse *I_Q_*(*t* − *t_n_*) decreases following the single impulse function. The single impulse function represents the two-compartmental models including the distribution and elimination function. We deliberately used the single input function calculated from the in vivo data of the oral formulations, because we aimed to avoid the influence of study-to-study variabilities. The arbitrary impulses *I_Q_*(*t*) are calculated by determining the in vitro partitioning rate every three minutes where the concentration in the organic layer was measured (Figure 3). The in vivo estimated gastric emptying of 30 min [16] was considered in the BiPHa+ assay by a pH shift from 1.0 to 5.5. We applied the time shift approach, i.e., only data where distribution into the organic phase were considered, in analogy with the methodology used in other research studies, which do not consider this 30 min lag-time for calculating IVIVR or pharmacokinetic prediction [6,7,8].

(2) $$C\left(t\right)=I_{Q}\left(t\right)*\;C_{\delta }\left(t\right)=\sum^{\;}I_{Q}\left(t-t_{n}\right)-A\;\cdot C\;{\left(t-t_{n}\right)}_{distribution}-B\cdot C\;{\left(t-t_{n}\right)}_{elimination}$$

To assess the prediction accuracy of the calculated pharmacokinetic profiles based on retrospective IVIVR, prediction errors (PE) according to EMA and FDA guidelines [1,2] of AUC (area under the curve of plasma profile), *C_max_* (maximum plasma concentration), and *t_max_* (time point with maximum plasma concentration) were calculated. All calculations were performed using EXCEL 2016 (Microsoft, Redmond, WA, USA).

(3) $$\mathrm{PE}\;[\%]=\frac{\mathrm{Observed}\;\mathrm{value}\;-\;\mathrm{Predicted}\;\mathrm{value}}{\mathrm{Observed}\;\mathrm{value}}$$

## 3. Results and Discussion

### 3.1. Drug Properties

The investigated drugs were characterized in terms of their LogP and pKa as well as aqueous solubility at gastric and intestinal pH (Table 3).

Celecoxib exhibits no physiological relevant pKa-value. pKa-values of the weak bases aprepitant, itraconazole, nimodipine and ritonavir are in the range of 1.9 to 3.8. Log P values were in the range of 3.5 to 5.4 (Table 3). The solubility was investigated in 0.1 M hydrochloric acid, phosphate buffer 6.8 and Bi-FaSSIF-V2 [14]. Itraconazole, ritonavir and aprepitant exhibit pH-dependent solubility. The solubility of celecoxib, aprepitant, nimodipine and ritonavir was up to 10-fold higher in the presence of sodium-taurocholate and lecithin (Bi-FaSSIF-V2 medium) compared to the surfactant-free dissolution media. According to the solubility values of the pure drugs, especially for those in the Bi-FaSSIF-V2 medium (Table 3), the aqueous dissolution takes place under non-sink conditions for all investigated drug substances. However, the solubility of the drug substances in the absorption (organic) phase of 1-decanol should be above 8.0 mg/ml (Table 1) to ensure 10-fold absorption sink-conditions in the organic phase during the BiPHa+ dissolution experiments. Except from itraconazole and aprepitant, the solubility of all other drugs was higher than 8.0 mg/ml. Itraconazole showed a solubility of 1.23 mg/ml in 1-decanol. Therefore, the volume of 1-decanol was adjusted to 100 ml to ensure a 5-fold sink. The solubility of aprepitant in 1-decanol was 4.2 mg/ml, which was assumed to be sufficient to ensure sink conditions (Table 3).

The absolute oral bioavailability was taken from literature (see Section 2.5, Table 4). The fraction absorbed (fa) and bioavailability for nimodipine are different, because 90% of the absorbed nimodipine is metabolized by first pass, which leads to a distinctly lower bioavailability [34].

### 3.2. Dose Assessment

**Figure 4:**
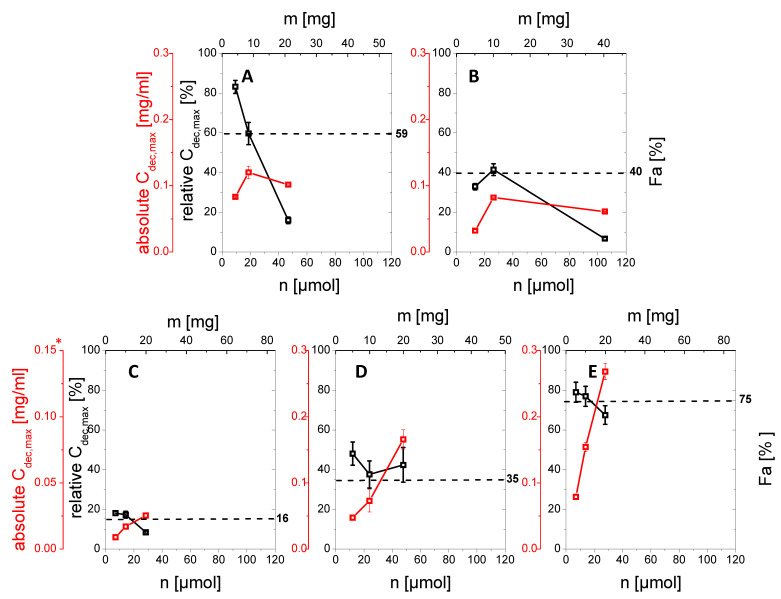
Relative *C_dec,max_* (%) and absolute *C_dec,max_* (mg/mL) values of the organic partitioning profiles with different amounts of drug (mg or µmol) investigated in the BiPHa+ assay after 4.5 h: (**A**) aprepitant; (**B**) celecoxib, (**C**) itraconazole *, (**D**) nimodipine; (**E**) ritonavir; dotted black line represents the in vivo oral fraction absorbed (%). * For keeping the decanol sink above 5 fold, itraconazole assays were carried out with 100 mL of decanol instead of 50 mL.

Each drug product was investigated at predetermined in vitro doses (5 mg, 10 mg and a BCS class dependent dose, see Section 2.4) by comparing the highest drug concentration in the organic layer (relative *C_dec,max_*) with the corresponding human fraction absorbed in vivo (fa(%), Table 4). The investigated in vitro doses of each formulation (µmol and mg) were plotted against the relative *C_dec,max_* (%; relative drug quantity partitioned in the organic layer at the end of the experiment regarding the investigated drug quantity of 5 mg, 10 mg, and BCS dependent) and the absolute molar partitioned quantity (µmol). Each relative *C_dec,max_* in the organic layer was directly assessed for biorelevance by comparing with the fraction absorbed in vivo (Figure 4).

Aprepitant (Emend^®^, Nanocrystal)

The drug product Emend^®^ contains the active aprepitant as nanocrystals. By increasing the in vitro dose in the BiPHa+ dissolution test from 5 mg (100%, 9.4 µmol) to 25 mg (100%, 46.8 µmol), relative *C_dec,max_*, in the organic layer decreased from 85% (4.25 mg, 7.8 µmol) to 18% (4.5 mg, 8.5 µmol) respectively.

The decrease was more pronounced in the dose range of 10 mg to 25 mg resulting in a relative *C_dec,max_* ranging from 60% (6.0 mg, 11.3 µmol) to 18% (4.5 mg, 8.5 µmol), compared to 5 and 10 mg (85–60%). Relative *C_dec,max_* at 10 mg was in good agreement to the fraction absorbed in vivo of approximately 59% (Figure 4A).

Celecoxib (Celebrex^®^, Microcrystal)

In the case of the micronized celecoxib formulation, the highest relative *C_dec,max_* value (41%, 4.1 mg) was achieved at a dose of 10 mg (100%, 26.2 µmol), which was comparable to the fraction absorbed in vivo. At 5 mg (100%, 13.1 µmol) celecoxib the relative *C_dec,max_* value was 32% (1.6 mg, 4.2 µmol). A lower relative amount of celecoxib (5%, 2.0 mg) partitioned into the organic layer at the BCS dependent in vitro dose of 40 mg (Figure 4B).

Itraconazole (Sempera^®^, ASD)

The enabling formulation of itraconazole is a HPMC-based amorphous solid dispersion, coated on pellets. Itraconazole partitioned up to 17% (1.3–2.4 µmol, 0.9–1.7 mg) in the case of the 5 mg (7.1 µmol) and 10 mg (14.2 µmol) investigated in vitro dose into the organic phase. 9% (2.6 µmol, 1.8 mg) of the 20 mg (28.3 µmol) itraconazole dose partitions in the organic phase. The in vivo fraction absorbed of the same formulation in the fasted state is 16% (Figure 4C).

Nimodipine (Nimotop^®^, ASD)

**Figure 5:**
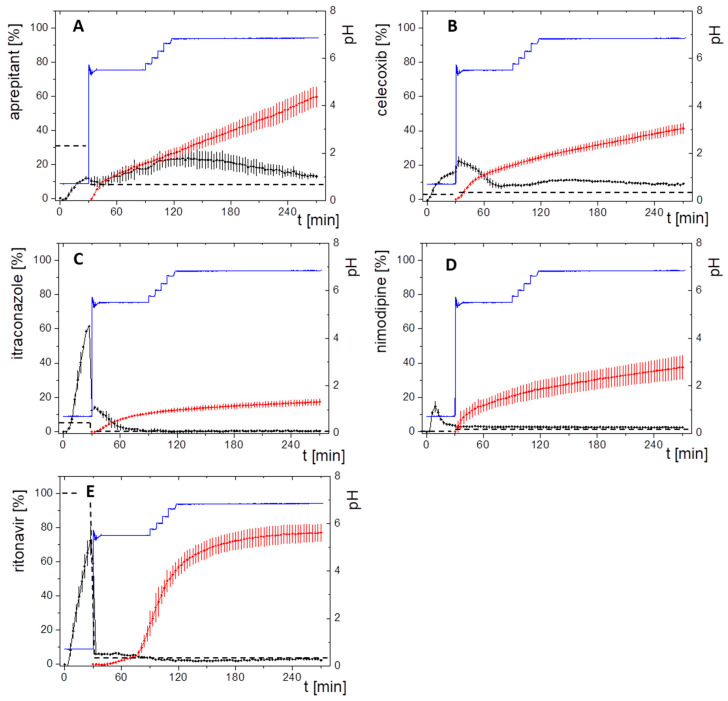
Biphasic dissolution profiles at 10 mg in vitro dose strength for (**A**) aprepitant; (**B**) celecoxib, (**C**) itraconazole; (**D**) nimodipine; (**E**) ritonavir. Drug concentration in the aqueous layer (black line), and in the organic layer (red line); pH in the aqueous layer during dissolution test (blue line); crystalline drug solubility in the aqueous layer (black dotted line).

Nimotop^®^ is a first generation ASD based on polyethylene-glycol. *C_dec,max_* values of 5 mg (11.9 µmol), 10 mg (23.9 µmol) and 20 mg (47.8 µmol) nimodipine-ASD were 50%, 38%, and 43% (5.7, 9.1 and 20.1 µmol; 2.5 mg, 3.8 mg and 8.6 mg), which was higher compared to 35% in vivo fraction absorbed (Figure 5D).

Ritonavir (Norvir^®^, ASD)

The Norvir^®^ drug product contains ritonavir as an amorphous solid dispersion consisting of a polymer/surfactant matrix. All investigated in vitro doses provided relative *C_dec,max_* of 80%, 77% and 70% (equal to 5.5, 10.6 and 18.6 µmol; 4.0, 7.7 and 17.5 mg) in the organic layer, which was in accordance with the in vivo fraction absorbed of 76% (Figure 4E).

The drug products with crystalline drug substance, namely Emend^®^ (aprepitant nanocrystals) and Celebrex^®^ (celecoxib microcrystals), showed similar in vitro behavior: an increase of the in vitro dose in the dissolution experiment led to decreased values for relative *C_dec,max_* in the organic layer. This behavior can be explained by the developability classification system [41,42] applied to the BiPHa+ assay. Dissolution of the crystalline drug at an in vitro dose level of 10 mg (15–25 µmol) was mainly dissolution rate limited (IIa) leading to a similar relative *C_dec,max_* in the organic layer. At the higher BCS class dependent dose levels as displayed in Table 1, drug dissolution became mainly solubility limited (IIb) leading to lower absolute *C_dec,max_* values (relative *C_dec,max_* stayed on the same level). As a result, the BiPHa+ assay was not able to discriminate the rate limiting step of dissolution at drug levels higher than 30 µmol (BCS dependent level), because the drug flux was limited by its solubility.

Ritonavir, itraconazole and nimodipine formulated as an ASD reached rather comparable relative *C_dec,max_* values in the 1-decanol layer over the tested in vitro dose levels. This could be explained by the fact that the three drugs form precipitates under the present experimental conditions during the intestinal stage. The partitioning into the organic layer did not only depend on solubility, because solubility severely drops in the intestinal stage. As described in the literature, there are many complex processes, which can occur simultaneously such as dissolution, precipitation and re-dissolution [8,13,43].

Relative *C_dec,max_* values obtained at an empirically determined in vitro dose of 10 mg for all investigated drugs demonstrated the highest accordance to the values for the fraction absorbed in vivo. Based on these results, we propose an in vitro dose of 10 mg (equal to approximately 15–25 µmol) for our assay to determine biocomparable partitioning profiles for in vivo doses in the investigated range of 30–200 mg. As (re-)dissolution, precipitation in the aqueous non-sink phase and partitioning into the organic sink are intended to be scalable to in vivo [23], we assume that the proposed settings are in this range. Further work is needed to evaluate in more detail the relationship between the scaling of the in vivo administered dose and the in vitro dose.

### 3.3. Drug Product Evaluation Using BiPHa+ Dissolution Assay

Five different market drug products formulated through different enabling principles were investigated under the same biorelevant biphasic dissolution setting mimicking fasted state conditions with a concentration of 10 mg in 50 ml aqueous phase (Figure 5). The equivalent molar and mass quantities are given in Table 1.

Aprepitant (Emend^®^, Nanocrystals)

In the aqueous phase the nanocrystal formulation provided an aprepitant dissolution of up to 15% in the gastric stage. The quantification of aprepitant within the intestinal stage became challenging, as pronounced scattering occurred from nano-sized particles in combination with Bi-FaSSiF-V2 surfactant (Figure 5A). Consequently, the quantification in the aqueous phase was probably overestimated leading to the curved shape of the aqueous concentration time profile. The partitioning rate into the organic layer represented a zero-order kinetic after a short phase of faster onset caused by the pH-dependent solubility. As evident from a relative *C_dec,mac_* of 60%, the dissolution of nanocrystals was very efficient.

Celecoxib (Celebrex^®^, Microcrystals)

The microcrystal formulation of the neutral drug celecoxib provided a drug dissolution in the gastric stage of up to 18% (Figure 5B). In the early intestinal stage (pH 5.5, Bi-FaSSIF-V2) additional drug dissolved up to 22% after 30 min in the aqueous phase. The initially higher partitioning rate into the organic layer was attributed to the higher concentration of the drug in the aqueous phase within the time span of 30–80 min. During the late intestinal phase, starting at 80 min, the aqueous concentration of celecoxib remained at a constant level of 8%, and thus results in a constant portioning rate.

Itraconazole (Sempera^®^, ASD)

Likely, the HPMC-based ASD enabled supersaturation of itraconazole in the aqueous gastric stage, which dissolves up to 60% at the 10 mg in vitro dose in the aqueous phase (Figure 5C). After the shift from gastric conditions to small intestine conditions, itraconazole precipitated resulting in approximately 17% remaining dissolved drug at minute 30. The short period of supersaturation in the early intestinal stage (30–60 min) was mainly responsible for the partitioned amount of itraconazole in the organic layer. After the supersaturation period (30–60 min), the precipitated drug was apparently no longer able to re-dissolve (60–270 min), which resulted in no further increase of the partitioned itraconazole (Figure 5C).

Nimodipine (Nimotop^®^, ASD)

In the case of the Nimotop^®^ drug product (Figure 5D), nimodipine dissolved from the PEG-based ASD and reached a maximum concentration in the aqueous phase of 5% within the first 15 min of the gastric stage. Precipitation occurred in the gastric stage during minutes 15 and 30. Even after a change to the intestinal stage, the nimodipine concentration did not exceed 5% in the aqueous phase until the end of the dissolution experiment after 270 min. The organic partitioning profile followed a square root-t ($\sqrt{t}$) kinetic. The relatively high *C_des,max_* in the organic phase was probably achieved by a high but decreasing re-dissolution rate of the precipitated drug.

Ritonavir (Norvir^®^, ASD)

Ritonavir exhibited a pH-dependent solubility. The ASD formulation provided drug dissolution in the aqueous phase of up to 75% within the first 30 min at gastric conditions. After the change to intestinal conditions (pH 5.5, FaSSIF-V2), the amount of dissolved ritonavir drops to 3%, and remained at this level until reaching the end of the dissolution experiment at 270 min. The drug concentration profile in the organic layer had a sigmoidal shape with a plateau at approximatelt 75% (Figure 5E).

After description of the results of the BiPHa+ dissolution experiments for each drug product, the following paragraphs discuss the results with respect to specific drug and formulation properties.

Crystalline Formulations

Aprepitant and celecoxib dissolved slowly in the gastric medium up to a concentration of less than 20%, because the drugs are formulated using their crystalline forms. The partitioning profile of the two crystalline drugs aprepitant and celecoxib followed a zero-order partitioning kinetic, because the drugs continuously dissolved from the crystalline particles released by the formulation, and only drug-specific solubility effects driven by pH and bile-salts influenced the extent of drug dissolution (Figure 5A–B).

Although the partitioning of the nano-sized aprepitant crystals was the fastest, conclusions on the influence of the crystal particle size were difficult to make, as the solubility of the respective drugs in the various media were different.

The partitioning profile of celecoxib was directly attributed to the dissolved amount of drug in the aqueous phase. The partitioning rate decreased with decreasing aqueous concentration. A higher drug concentration than the saturation solubility was likely caused by enhanced dissolution of celecoxib in the presence of the surfactants and lipids in the biorelevant intestinal medium (Table 3) [44].

Amorphous Solid Dispersion

The drug dissolution mechanisms of the ASD-based formulations, responsible for the drug partitioning in the organic layer, were different (Figure 5C–E). Supersaturation, which was indeed responsible for an increase of the partitioning rate [45], was only observed in the case of itraconazole (Figure 5C). The partitioning of nimodipine and ritonavir was mainly driven by the ability of the precipitated drug to re-dissolve (Figure 5D,E) [8,21].

The ability of nimodipine to re-dissolve was likely characterized by the organic partitioning profile, which decreased over time. A possible explanation was reported by Raina et al.: nimodipine amorphously precipitated and then started to crystalize moderately fast, leading in a decreased re-dissolution and subsequent partitioning [46].

The partitioning profile of the ritonavir formulation resulted in a sigmoidal shape. This was caused by a dynamic behavior of the drug-rich nano-droplets. The rather small particles underwent particle size reduction by re-dissolution. A plateau was reached as soon as all small drug-rich nano-droplets were dissolved [13].

Neutral Drugs

As pH-dependent solubility did not apply for neutral drugs, surfactant-mediated solubility enhancement could be observed even more pronounced for celecoxib in combination with the biorelevant surfactant from Bi-FaSSiF-V2. In our biphasic assay this surfactant sensitivity resulted in initially higher partitioning rates of celecoxib into the organic phase related to dissolved/solubilized drug (Figure 5B). Celecoxib showed chaser properties, whereby the micronized formulation supersaturated, i.e., next to undissolved crystalline celecoxib a supersaturated solution was present. Despite crystalline celecoxib being present, the supersaturated amorphous solubility remains, because the precipitated celecoxib slowly recrystallized [47].

pH-Dependent Soluble Drugs

The expected pH-dependent solubility of the weak base aprepitant is barely noticeable (Figure 5A). Consequently, the particle size reduction approach of the crystalline aprepitant was the main reason for the increased dissolution and in turn the high partitioning rate. However, the solubility of the weak bases ritonavir and itraconazole was strongly pH –dependent: The dissolved amount of both drugs dropped remarkably after entering the early intestinal stage at elevated pH. At the solubility maximum at pH 1, itraconazole initially showed supersaturation followed by precipitation over a period of 30 min. The generated supersaturation was responsible for itraconazole partitioning in the organic layer. Once the crystalline solubility was re-established, itraconazole had no further tendency to re-dissolve. In contrast, ritonavir formed nano-droplets generated by liquid–liquid phase separation resulting in a constant aqueous concentration, and a highly increased partitioning rate [13].

Although nimodipine has a measured pKs value of 2.6, it precipitated already during the gastric phase (pH = 1.0). The change of partitioning rate was likely driven by the properties of the amorphously precipitated nimodipine [46]. This can be seen by the fact that the dissolved nimodipine concentration remained on the same level and the partitioning rate changed.

### 3.4. Pharmacokinetics and Compartment Analysis of In Vivo Data

As described in Section 2.5, distribution (λ_1_) and elimination (k_e_) were calculated from in vivo data (Table 5) by a two-compartment model (Figure 3), due to the high LogP values of the investigated drugs [26]. The high lipophilicity results in high protein binding distribution into tissue [26]. Since aprepitant has a more complex elimination profile, it was pragmatically described by a one-compartment model [28].

The pharmacokinetic constants were calculated using the residual method based on literature data (Table 5). The estimated absorption rate constant (k_a1_) of the drugs aprepitant, celecoxib, itraconazole and ritonavir were quite comparable between 1.1 h^−1^ and 1.6 h^−1^. The determined absorption rate of nimodipine is also very fast with a rate constant of 2.0 h^−1^. However, a substantial part of the absorbed dose subsequently undergoes a high first-pass hepatic metabolism. As the metabolism rate of nimodipine is higher than the absorption rate, it could not be calculated based on the available data [24].

These findings demonstrated the applicability of the compartment model combined with the residual method to get a meaningful estimate of the absorption rate by a first order kinetic.

### 3.5. Verification of In Vivo Relevance and Predictive Power

#### 3.5.1. IVIVR: Compare Drug Partitioning during Dissolution and In Vivo Absorption

**Figure 6:**
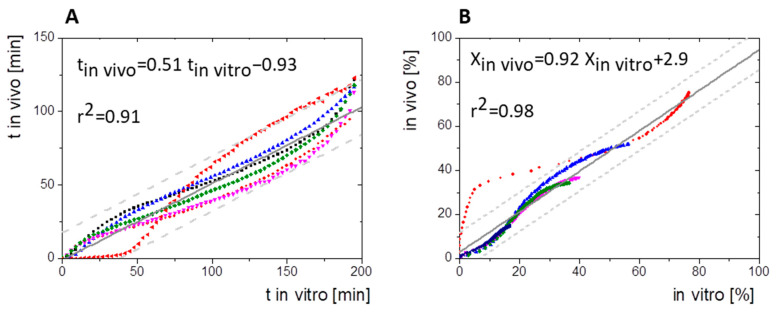
(**A**) Levy Plot/ time-scaling; time to have X% absorbed in vivo vs. partitioned in vitro without lag-time, (**B**) IVIVR of the five model drugs without lag-time both including confidence interval 95% (grey dashed lines), aprepitant (blue symbols), celecoxib (pink symbols), itraconazole (dark blue symbols), nimodipine (green symbols), red: ritonavir (red symbols).

Because the absolute bioavailability values were comparable to the end-concentrations of the organic layer relative *C_max,dec_* (Figure 4), a level A IVIVR was established based on the obtained in vivo absorption data and the results of the BiPHa+ dissolution test. Two cases were considered to assess the correlation between in vitro drug partitioning into the organic layer and the fraction absorbed in vivo:

In the first case, a regular level A IVIVR was calculated based on the Levy plot to commonly simulate the time-scaling between in vitro dissolution and in vivo absorption data (Figure 6A,B). The correlation of the Levy plot for all model drugs resulted in a regression coefficient of 0.91. Most single time values were within the 95% confidence interval (Figure 6A). The resulting fractions absorbed were plotted against the drug concentration in the organic layer obtained during the in vitro dissolution, which led to a regression coefficient of 0.98 (Figure 6B). However, correlation values of ritonavir were outside the 95% prediction limit (Figure 6B). All IVIVR data points are sigmoidal distributed except those for ritonavir. This is caused by rapid initial partitioning rates compared to the calculated first order absorption profiles, which is reflected on the IVIVR plot by data points placed below the regression line within the first 20% in vitro partitioned drug (Figure 6B). Then, the partitioning rates decrease and the IVIVR data points distribute above the regression line. A profound interpretation of this finding is hardly possible, since the true in vivo absorption is not known.

**Figure 7:**
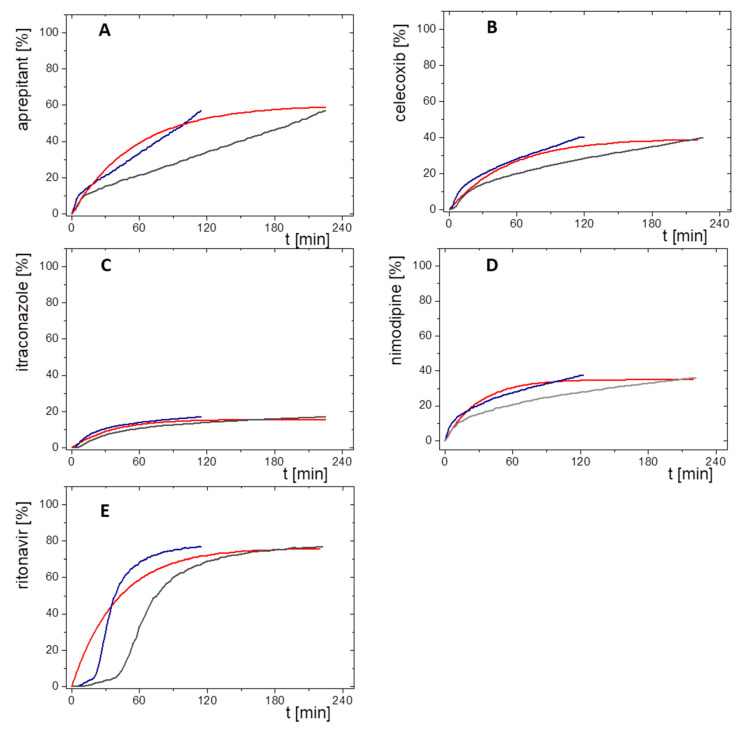
Comparisons of in vivo absorption profiles (%) and relative in vitro partitioning profiles of the 10 mg in vitro dose (%) plotted against time (min) without lag-time for (**A**) aprepitant; (**B**) celecoxib, (**C**) itraconazole; (**D**) nimodipine; (**E**) ritonavir; red line: fraction absorbed time profile in vivo; blue line: relative organic partitioning profiles corrected by Levy plot (t(Levy) = t × 0.51); grey line: untreated relative organic partitioning profiles.

In the second assessment, a direct comparison between in vivo absorption and in vitro partitioning profiles was performed (Figure 7). In consideration of the absolute bioavailability, the calculated first order absorption profiles were compared to the in vitro partitioning profiles. Both the directly measured (grey line) and the time-scaled (blue line, Levy plot) in vitro partitioning profiles are given in Figure 7. Levy plots are generally implemented, when rate constants of drug absorption and in vitro dissolution are different [39]. However, in our cases the rate constants of the BiPHa+ assay were similar with the in vivo constants. The partitioning profiles with time-scaling (blue lines, Figure 7) matched the calculated in vivo fraction absorbed profiles better than the not time-scaled partitioning profiles (grey lines, Figure 7). However, similarities of the time-scaled partitioning and absorption profiles reflect the result of the Levy plot and are not necessarily of physiological relevance. Since the end concentration of the partitioned drug (relative *C_dec,max_*) in the organic phase matched very well with the fraction absorbed by using the 10 mg in vitro dose (Figure 7). Both data sets were used for the pharmacokinetic predictions. Because the same relative *C_dec,max_* values were used for the predictions considering the time-scaled and not time-scaled partitioning profiles, the plasma concentration time profiles differ in shape but not in the resulting AUC.

Overall, the relative partitioned amount of the investigated poorly soluble drug formulations was equivalent to the maximum amount of passively absorbed drug fraction in vivo.

#### 3.5.2. In Vivo Prediction from Biphasic In Vitro Data and Prediction Error

**Figure 8:**
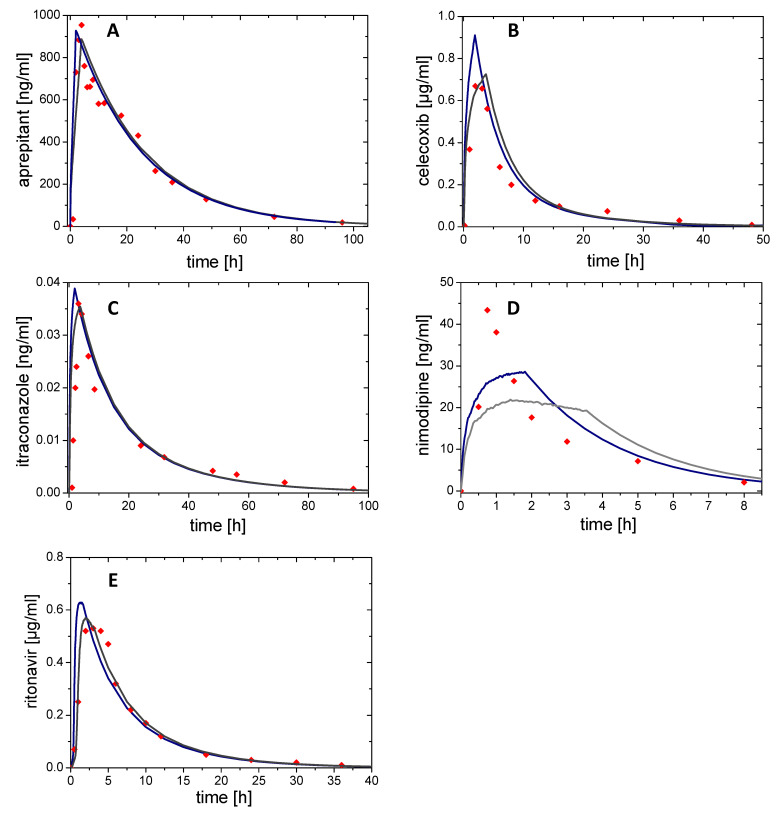
Plasma concentration time profiles for (**A**) aprepitant, (**B**) celecoxib, (**C**) itraconazole, (**D**) nimodipine, and (**E**) ritonavir. Modelled in vivo profile with time-scale correction (blue line); Modelled profile without time scale correction (grey line); Plasma concentration time profiles from humans (red dots).

The information on distribution and elimination obtained from the compartmental models (derived from the published in vivo data) was combined with the corresponding partitioning profile obtained from the BiPHa+ dissolution experiment Equation (2) to calculate the plasma concentration time profiles in vivo. Figure 8 shows the predicted in vivo plasma concentration time profiles without (grey line) and with (blue line) time-scaling (Levy plot). The calculated pharmacokinetic profiles are compared to the observed plasma concentrations. Pharmacokinetic parameters and prediction errors of observed and predicted plasma concentration time profiles are given in Table 6.

Aprepitant (Emend^®^, Nanocrystals)

In case of aprepitant, the applied one compartment model was in good agreement with the observed in vivo data (Figure 8A). By using the concentration time profile from dissolution (organic layer) without time-scaling as absorption profile, a C_max_ of 0.89 µg/ml was calculated to be reached at 3.9 h (t_max_), which is in a good agreement with the reported t_max_ in vivo (4.1 h) as a result of the zero-order dissolution kinetics in vitro. On the other hand, the use of the time-scaled in vitro dissolution profile led to a similar C_max_ value of (0.93 µg/ml) but an essentially lower t_max_ (2.0 h) (Figure 8A).

Celecoxib (Celebrex^®^, Microcrystals)

After an initial increased partitioning rate, celecoxib partitioned with a zero-order kinetic in the dissolution experiment. This partitioning profile was used as basis for the absorption kinetic and led to the tip shape of the mathematically predicted pharmacokinetics (Figure 8B). T_max_ of the predicted time scaled profile (1.9 h) was closer to the observed in vivo data (2.0 h) than the t_max_ calculated without time-scaling (3.8 h). Overall, the predicted AUC was in good agreement with the reported AUC value for celecoxib (Table 6). The absolute fraction absorbed was estimated to be in the range of 40% by applying the present IVIVR model in absence of human plasma concentration data after intravenous dosing.

Itraconazole (Sempera^®^, ASD)

The tip shape of the itraconazole plasma profile and the predicted profiles (Figure 8C) were the result of the fast absorption in vivo (Figure 7C). Compared to the absorption profiles in vivo the partitioning profiles in vitro had similar shapes (Figure 7C), and demonstrated the high accuracy of the predicted plasma concertation time profiles (Figure 8C). Both concentration time predictions, with and without time-scaling, were only slightly different to the in vivo observed data (Table 6).

Nimodipine (Nimotop^®^, ASD)

The predicted plasma concentration time profile of the nimodipine drug product showed the highest prediction error in C_max_ of 32.5% and 48.9% (Table 6). Due to the high extent of first-pass [35] and the high elimination rate, describing pharmacokinetics accurately was difficult. In fact, enzyme saturation occurs in vivo [35], which was of course not covered by the applied two-compartmental models (Figure 8D). As the absorption rate is slower than the metabolizing rate, determining a metabolizing rate by in vivo plasma data was not possible. The only option to consider the metabolism in this case would be using the bioavailability (F = 3.5%) in the prediction derived from the in vivo fraction absorbed (f_a_ = 35%). Due to the short elimination half-life period, the partitioning profile strongly influences the shape of the pharmacokinetic prediction profile (Figure 8D).

Ritonavir (Norvir^®^, ASD)

The in vivo performance of ritonavir could be very precisely predicted (Figure 8E). The sigmoidal shape of the in vitro dissolution profile led to a lag-time in the predicted profiles (Figure 5E). Due to the faster partitioning rate of the in time-scaled prediction, C_max_ (0.63 µg/ml) was overestimated and the not time-scaled (0.56 µg/ml) was slightly overestimated (Table 6).

Finally, based on the modelling results, prediction errors for AUC and *C_max_* were calculated and assessed based on the guidance from EMA and FDA [1,2]. In addition, the deviations of *t_max_* were determined as absolute values in hours. The results are displayed in Table 6, and summarized as follows:

The average prediction error of AUC of time scaled corrected and not corrected partitioning profiles was (+) 0.24%, which confirmed the accuracy of the prediction. Furthermore, the prediction error for the AUC of single drug plasma concentration time profile was in the acceptance range of 15% [11].

The average mean prediction error was (−) 5.7% for *C_max_* based on time-scaled plasma concentration time profile, and (+) 8.6% without time-scaling.

The mean deviation of *t_max_* of the predicted plasma concentration time profiles from the available pharmacokinetic data reported in the literature is less than one hour (−0.8 h and +0.4 h). All *t_max_* values do not deviate from −2.1 h to +1.8 h for more than two hours.

In sum, the BiPHa+ assay delivered meaningful, in vivo relevant partitioning profiles of different enabling formulations. First, a level A IVIVR was successfully established for all formulations, providing an accurate estimate of the absolute fraction absorbed based on in vitro data using a single set of assay parameters.Second, the in vivo performance was successfully predicted based on compartmental models. Deviations from the observed in vivo data can be explained by metabolic pathways of the respective drug. A time-scaling step might be not essential, because the Levy plot just minimizes deviations of the in vitro partitioning profiles from the first order absorption kinetic in vivo and both fraction absorbed and the highest relative partitioned drug quantities (relative *C_dec,max_*) were in the same range at 4.5 h (the end of BiPHa+ assay).

Third, the prediction errors of predicted plasma concentration time profiles were in good agreement with EMA and FDA IVIVC guidelines. The higher prediction errors of *C_max_* compared to AUC are due to the mathematic prediction of plasma concentration time profiles, and are related to specific drug pharmacokinetic properties, such as the strong first-pass effect and hepatic metabolism, which are not reflected by the BiPHa+ assay. In this case physiological based pharmacokinetic modelling would be appropriate to predict plasma concentration time profiles based on in vitro data.

## 4. Conclusions

The goal of the current study was to investigate the in vivo relevance of the BiPHa+ biphasic dissolution assay using a single, general set of test parameters, in which five drug products were investigated, each drug formulated by a different enabling approach (micro- and nanocrystals, amorphous solid dispersion).

The mini-scale approach of the BiPHa+ test system required a down-scaling of the drug dose level. It was found that 15–25 µmol (~10 mg) of drug was a meaningful in vitro dose to reach high in vivo relevance for dose ranges of the tested drug products (30–200 mg). The correlation indicated that in this dose range the in vitro concentration time profiles of the partitioned drugs in the organic layer potentially behaved similar to the passively absorbed drug amount in vivo (3.2).

The in vitro concentration time profiles of the partitioned drugs in the organic layer (3.3) were successfully correlated with their in vivo fraction absorbed concentration time profiles obtained from published human data (3.5).

In most cases of the highly lipophilic drugs, a two-compartmental model was suitable to predict the in vivo pharmacokinetics calculated based on the in vitro partitioning profile from various enabling formulations (3.5). As soon as a high degree of metabolism is present, predictions are difficult, since the BiPHa+ assay characterizes rather absorption. For a more precise description, saturable metabolic mechanisms would be necessary, which can be simulated by PBPK modelling approaches, for example.

Overall, the results indicate that the rationally developed BiPHa+ assay with pH-shift is capable to deliver biorelevant partitioning profiles. Establishing a shared IVIVR using the compartmental prediction approach is a valuable tool to assess the biphasic model in terms of in vivo relevance. Consequently, the BiPHa+ assay is an in vivo relevant characterization tool for enabling formulations.

## Figures and Tables

**Table 1:** Investigated drug products: Drug substance, trade name, highest dose strength on market, and enabling formulation type. A dose strength of 10 mg was used for further biorelevance investigations (marked in bold); BCS related in vitro doses based on highest dose strength divided by 250 mL are marked in italics.

Drug Substance	Trade Name	Formulation Type	Investigated In Vitro Dose **		
	(Dose Strength)		(mg)	(mg/mL)	(µmol)
Aprepitant	Emend^®^ (125 mg)	Nanocrystal (Capsule, Pellets)	5	0.1	9.4
			**10**	***0.2***	**18.7**
			*25*	*0.5*	*46.4*
Celecoxib	Celebrex^®^ (200 mg)	Microcrystal (Capsule, Powder)	5	0.1	13.1
			**10**	***0.2***	**26.2**
			*40*	*0.8*	*104.8*
Itraconazole	Sempera 7^®^ (100 mg)	ASD (Capsule, Pellets)	5	0.1	7.1
			**10**	***0.2***	**14.2**
			*20*	*0.4*	*28.3*
Nimodipine	Nimotop^®^ (30 mg)	ASD (Tablet)	*5*	*0.1*	11.9
			**10**	***0.2***	**23.9**
			*20* *	*0.4* *	*47.8* *
Ritonavir	Norvir^®^ (100 mg)	ASD (Tablet)	5	0.1	6.9
			**10**	***0.2***	**13.9**
			*20*	*0.4*	*27.7*

* A 20 mg in vitro dose was additionally investigated. ** Used as tablet piece or powder/pellets taken out from the capsule for drug dissolution testing in the BiPHa+ apparatus.

**Table 2:** Overview of the methods applied for in vitro and in vivo data treatment. Each method compromises one or more operations as well as the required input and output data.

Method	Operation	Input	Output
Compartmental analysis	Residual Method (Compartmental analysis)	In vivo pharmacokinetic data	Absorption rate constant (profile) Distribution rate constant (profile) Elimination rate constant (profile)
In vitro/in vivo relationship	(1) Levy plot	In vitro partitioning profile (without time-scaling) Absorption rate constant (profile)	Time-scaling
	(2) Fraction absorbed vs. partitioned drug plot (IVIVR)	In vitro partitioning profile (with time-scaling) Absorption rate constant	IVIVR
Comparisons of in vivo absorption profiles and in vitro partitioning profile	n/a *	In vitro partitioning profile (with and without time scaling) Absorption rat constant (profile)	n/a *
Pharmacokinetic prediction	(1) Derivation of partitioning curve	In vitro partitioning profile (with and without time-scaling)	Arbitrary impulses
	(2) Residual Method (Compartmental analysis)	In vivo pharmacokinetic data	Single impulse response (Distribution rate constant & Elimination rate constant)
	(3) Convolution	Single impulse response Arbitrary impulses	Predicted pharmacokinetic
	(4) Prediction accuracy	Predicted pharmacokinetic In vivo pharmacokinetic data	Prediction errors

* not applicable.

**Table 3:** Physiochemical characterization of 5 investigated drugs: pKa values, solubility (S); LogP; bioavailability (F) and fraction absorbed (fa) and quantification wavelength (spectra processing method: 1.der: first derivative; exp: exponential correction; off: offset).

Parameter	Aprepitant	Celecoxib	Itraconazole	Nimodipine	Ritonavir
pKa	2.8	10.7 (acid)	3.8	2.6	1.9 2.5
S (0.1N HCl) (µg/mL)	62.0	2.67	6.1	3.21	382.8
S (6.8N Buffer) (µg/mL)	1.39	1.76	0.88	2.90	0.96
S (FaSSIF-V2) (µg/mL)	14.0	4.52	0.60	5.18	4.3
S (1-decanol) (mg/mL)	4.3	>8.0	1.23	>8.0	>8.0
Log P	4.8	3.7	5.4	3.5	4.4
Wavelength (in aqueous phase) (nm)	283 (1. der)	285 (1.der)	278 (1.der)	366 (exp)	240 (exp)
Wavelength (in 1-decanol) (nm)	280 (1. der)	268 (off)	290 (1.der)	366 (off)	263 (1.der)

**Table 4:** Bioavailability (F, considering first-pass) and fraction absorbed (fa, without first pass) reported in the literature.

Parameter	Aprepitant	Celecoxib	Itraconazole	Nimodipine	Ritonavir
Bioavailability (F)	0.59	0.39	0.16	*0.035*	0.75
	[27]	[29]	[32,33]	[25,26]	[8]
Fraction absorbed (fa)	0.59	0.39	0.16	*0.35*	0.75
	[27]	[29]	[32,33]	[25,26]	[8]

**Table 5:** Calculated pharmacokinetic rate constants of the in vivo human plasma profile: k_a1_ (absorption rate), k_a2_ (activation rate), λ_1_ (distribution rate), k_e_ (elimination rate).

Rate Constant	Aprepitant	Celecoxib	Itraconazole	Nimodipine	Ritonavir
$k_{a1}\;\left[h^{-1}\right]$	1.050	1.150	1.580	2.040	1.496
$\lambda_{1}\;\left[h^{-1}\right]$	N/A	0.257	0.0931	0.481	0.123
$k_{e}\;\left[h^{-1}\right]$	0.0421	0.070	0.0352	0.351	0.112

N/A: not applicable.

**Table 6:** Prediction errors for AUC, *C_max_* and *t_max_* based on FDA and EMA guidance [1,2] comparing time-scaled (Levy) dissolution profile and those without time-scaling.

Drug Product	Origin of Absorption Kinetic	AUC	Error AUC (%)	*C_max_*	Error *C_max_* (%)	*t_max_*	Error *t_max_*
		(µg/mL∙h)		(µg/mL)		(h)	(h)
Aprepitant	in vivo	22.5		0.95		4.1	
	in vitro (Levy) in vitro	20.7	8	0.93	2.1	2	−2.1
				0.89	6.3	3.9	−0.2
Celecoxib	in vivo	5.96		0.67		2	
	in vitro (Levy) in vitro	5.8	2.7	0.91	−35.8	1.9	−0.1
				0.73	−8.9	3.8	1.8
Itraconazole	in vivo	0.7		0.036		3	
	in vitro (Levy) in vitro	0.723	−3.0	0.039	−8.3	1.9	−1.1
				0.035	2.8	3.8	0.8
Nimodipine	in vivo	0.104		0.043		0.8	
	in vitro (Levy) in vitro	0.112	−7.7	0.029	32.5	1.7	0.9
				0.022	48.9	1.4	0.6
Ritonavir	in vivo	4.73		0.53		3	
	in vitro (Levy) in vitro	4.66	1.5	0.63	−18.9	1.4	−1.6
				0.56	−5.7	2.1	−0.9

## Data Availability

All data presented in this study are available in the research article.
